# LRRK2 in Parkinson's disease and dementia with Lewy bodies

**DOI:** 10.1186/1750-1326-1-17

**Published:** 2006-11-30

**Authors:** Xiongwei Zhu, Asim Babar, Sandra L Siedlak, Qiwei Yang, Genta Ito, Takeshi Iwatsubo, Mark A Smith, George Perry, Shu G Chen

**Affiliations:** 1 Department of Pathology, Case Western Reserve University, Cleveland, Ohio, USA; 2 Department of Neuropathology and Neuroscience, University of Tokyo, Tokyo, Japan; 3 College of Sciences, University of Texas at San Antonio, San Antonio, Texas, USA

## Abstract

**Background:**

Mutations in *LRRK2 *encoding leucine-rich repeat kinase 2 are thus far the most frequent genetic cause associated with autosomal dominant and idiopathic Parkinson's disease (PD). To examine whether LRRK2 is directly associated with neuropathology of PD and other related disorders, we analyzed LRRK2 in brains of patients affected by PD and dementia with Lewy bodies (DLB) using highly specific antibodies to LRRK2.

**Results:**

We demonstrated that anti-LRRK2 antibodies strongly labelled brainstem and cortical Lewy bodies, the pathological hallmarks of PD and DLB, respectively. In addition, anti-LRRK2 also labelled brain vasculature, axons, and neuronal cell bodies. Interestingly, the immunocytochemical profile of LRRK2 varied with different antibodies depending upon specific antigenic sites along the LRRK2 protein. All anti-LRRK2 antibodies tested that were raised against various regions of LRRK2, were found to be immunoreactive to recombinant LRRK2 on Western blots. However, only the antibodies raised against the N-terminal and C-terminal regions of LRRK2, but not the regions containing folded protein domains, were positive in immunolabeling of Lewy bodies, suggesting a differential exposure of specific antigenic sites of LRRK2 on tissue sections.

**Conclusion:**

We conclude that LRRK2 is a component of Lewy bodies in both PD and DLB, and therefore plays an important role in the Lewy body formation and disease pathogenesis. Information on the cellular localization of LRRK2 under normal and pathological conditions will deepen our understanding of its functions and molecular pathways relevant to the progression of PD and related disorders.

## Background

Parkinson's disease (PD) is the most common neurodegenerative disorder of the extrapyramidal system, affecting up to 3% of individuals aged 65 years and older and over 1 million people in North America [1,2]. The disease is characterized clinically by resting tremor, rigidity, bradykinesia, and postural instability and pharmacologically by response to l-dopa treatment. Neuropathologically, it is associated with selective loss of dopaminergic neurons and the presence of intracytoplasmic proteinaceous inclusions, known as Lewy bodies (LBs), in the substantia nigra [1]. The etiology and pathogenesis of PD remains enigmatic, but growing evidence has suggested a genetic basis for PD. Mutations in genes encoding parkin, PTEN-induced putative kinase 1, and DJ-1 are segregated with autosomal recessive, early-onset familial PD, whereas those in genes encoding α-synuclein and leucine-rich repeat kinase 2 (LRRK2) are linked to autosomal dominant PD [3-5]. Of these five genes, only LRRK2 mutations have been shown to be both a common cause and a risk factor for familial PD and sporadic PD, respectively. LRRK2 mutations accounted for up to 6–40% of familial PD cases depending on the ethnic populations studied, and up to 2% of community-base sporadic PD cases [6-11].

The clinical and pathological features associated with the LRRK2 mutations are in most cases consistent with those of late-onset PD but may vary depending on the type of pathogenic mutations. It has been reported that patients carrying the most common G2019S mutation present a disease phenotype that is indistinguishable from idiopathic PD, which is characterized by classical Lewy body pathology [12-14]. Instead, patients carrying the less common R1441C and Y1699C mutations may have a more variable disease presentation that can include PD and other clinical parkinsonism such as dementia with Lewy bodies (DLB) and progressive supranuclear palsy [4]. These apparently pleomorphic pathologies may reflect the effect of specific pathogenic substitutions on the structure and function of LRRK2.

The neuropathological hallmark of PD is the presence of spherical LBs in affected brainstem. LB inclusions are also a prominent pathological feature of DLB, but are of more diffused nature and are found in cortical regions [15]. Among proteins genetically associated with PD, α-synuclein and parkin have been previously shown to be components of LBs in both PD and DLB [16-18]. Both α-synuclein and parkin are known to be phosphoproteins [19-21] and hence are potentially regulated by LRRK2 serving as a functional kinase [22,23]. It is therefore of particular interest to determine whether LRRK2 also localizes to LBs. Given that LRRK2 is such a large protein with 2527 amino acids and multiple protein domains, we performed a detailed analysis of LRRK2 localization by using a battery of antibodies against different regions spanning the entire protein to address this important issue. Here we provide strong evidence that LRRK2 is localized, both *in situ *and following isolation, to LBs in both PD and DLB.

## Results

### Protein biochemistry

**Figure 1:**
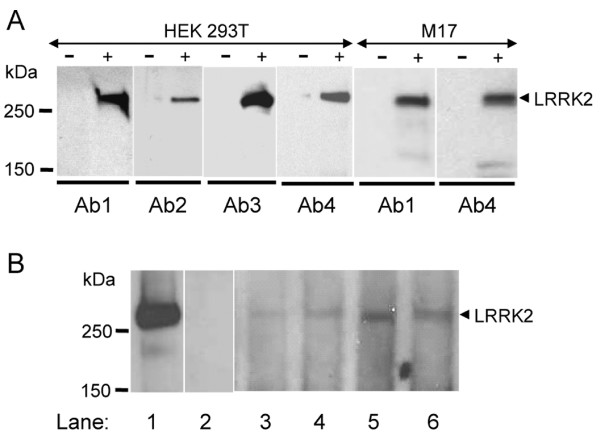
**Western blotting of LRRK2**. **A. **Recombinant LRRK2 (arrowhead) from transfected (+) HEK 293T and M17 cells was specifically recognized by all four LRRK2 antibodies (Ab1, Ab2, Ab3, and Ab4) used in this study. LRRK2 was not recognized in non-transfected cells (-). **B. **Recombinant LRRK2 (arrowhead) from transfected M17 cells is detected by anti-LRRK2 Ab4 (lane 1), but not after absorption of the antibody with its peptide antigen (lane 2). Brain LRRK2 was recognized by anti-LRRK2 Ab4 in two controls (lanes 3 and 4) and two PD cases (lanes 5 and 6). Cell lysates (10 μg protein) and brain homogenates (100 μg protein) were prepared and loaded on 6% SDS-PAGE gels for Western blot analysis using anti-LRRK2 antibodies Ab1–Ab4 in (**A**) and Ab4 in (**B**).

LRRK2 is predicted to be a large protein of 2527 amino acids with a complex composition including five consecutive domains; leucine-rich repeats (LRRs), Ras of complex protein (ROC), C-terminal of ROC (COR), mitogen-activated protein kinase kinase kinase (MAPKKK), and WD40 repeats [24]. To determine the expression and localization of LRRK2 in brain, four affinity-purified antibodies against LRRK2 were used for Western blotting and immunocytochemistry. These antibodies were raised against different regions of LRRK2 that span the entire protein (see Materials and Methods for details). The epitopes for Ab1 and Ab4 are located at the N-terminal and C-terminal to (and hence outside) the core protein domains of LRRK2, respectively, while those for Ab2 and Ab3 are located within the domains of LRRs and COR/MAPKKK, respectively. To characterize the immunoreactivity of these antibodies to LRRK2, we expressed human LRRK2 in HEK293T and M17 cells by transient transfection with a mammalian expression vector pCMV6-XL4/LRRK2. On Western blots, all four antibodies (Ab1, Ab2, Ab3 and Ab4) readily detected a prominent LRRK2 band migrated at about 280 kDa, the size expected for the full-length LRRK2, in lysates of transfected cells but not in non-transfected cells (Figure 1A). Thus, all four antibodies are highly specific for LRRK2 overexpressed in cultured cells. Both non-neuronal (HEK293T) and neuronal (M17) cell lines produced high levels of LRRK2 following transient transfection with its cDNA construct. The endogenous LRRK2 in non-transfected cells were not detected at the total protein level loaded (10 μg of lysates). Taken together, our results indicate that under denaturing conditions of SDS-PAGE, the epitopes of LRRK2 in different sites are fully exposed and can be readily recognized by corresponding antibodies on Western blots.

We have attempted to use these anti-LRRK2 antibodies to detect brain LRRK2 in subjects without neurological diseases and those with sporadic PD. Results from anti-LRRK2 Ab4 was presented here but similar data were obtained with other antibodies. As an additional control for the specificity of anti-LRRK2 Ab4, we performed the antigen absorption experiment in which the antibody was incubated with its corresponding peptide antigen prior to Western blotting. As shown in Figure 1B, the immunoreactivity to LRRK2 by anti-LRRK2 Ab4 was completely blocked following the absorption with its antigen, confirming that the antibody is highly specific for LRRK2. Brain LRRK2 was expressed at low levels but was detectable by using anti-LRRK2 Ab4 when 100 μg of total protein of brain homogenates were used. The LRRK2 bands from brain samples had same gel migration at 280 kDa as that from transfected cells, with little evidence of degradation or autolysis. Endogenous LRRK2 in brain homogenates from two control subjects and two PD patients was specifically recognized by anti-LRRK2Ab4 (Figure 1B). Overall intensity of the LRRK2 band was stronger in these PD samples as compared to the controls. In summary, LRRK2 from cultured cells and brain tissue can be specifically identified on Western blots by several different antibodies against a broad range of epitopes along the protein.

### Immunocytochemistry-PD

**Figure 2:**
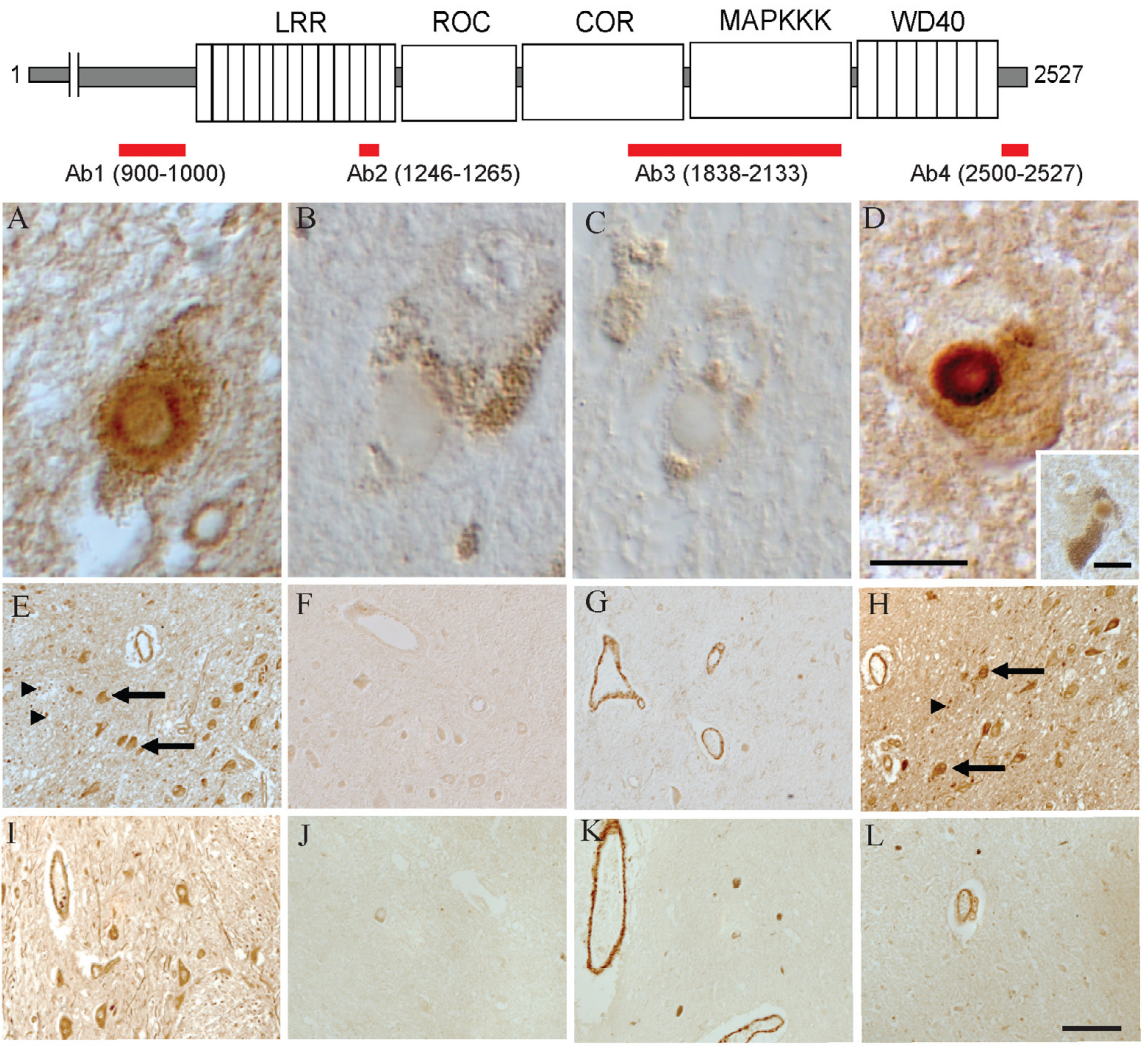
**Immunocytochemistry of LRRK2 in PD**. Four antibodies raised against sequences corresponding to various regions shown on the schematic diagram of LRRK2 (red bars) were used on brainstem sections of PD (**A-H**) and age-matched controls (**I-L**). Intense immunolabeling of brainstem LBs in cases of PD was seen with Ab1 against LRRK2_900–100 _(**A**) and Ab4 against LRRK2_2500–2527_(**D**). Both rim (**A **and **D**) and core (inset in **D**) of LBs were recognized. In contrast, LBs were not labelled in any case using antibodies directed against LRRK2_1246–1265 _(Ab2, **B**) or LRRK2_1838–2133 _(Ab3, **C**), for which the antigenic sites are located within the folded domains. The cell bodies of both pigmented and no-pigmented neurons (arrows) as well as axons (arrowheads) contain LRRK2, seen only using antibodies against sites outside the folded domains (Ab1, **E **and Ab4, **H**). In contrast, Ab4 staining was much less intense in control tissue (**L**). The muscle layer of both large and small vessels was consistently found to contain high levels of LRRK2 in almost all PD cases (seen in **E**, **G**, and **H**) and strikingly the vessels are the only structure immunolabeled with Ab3 recognizing LRRK2_1838–2133 _in all cases studied (**G **and **K**). Ab2 to LRRK2_1246–1265 _did not recognize LBs, cell bodies or vessels in any case (**B**, **F**, and **J**). Scale bars: **A-D **and inset = 10 μm; **E-L **= 100 μm.

All four antibodies were used to immunostain brainstem sections from six cases of PD and age-matched controls. Most notable was the finding that in all cases of PD examined, LBs in the brainstem were intensely labelled with Ab1 and Ab4 recognizing LRRK2_900–1000 _that is immediately N-terminal to the first LRRs domain and LRRK2_2500–2527 _at the extreme C-terminus, respectively (Figure 2A and 2D). Both rim and core of brainstem LBs were labelled by Ab1 and Ab4, with a similar pattern to that seen with an antibody to α-synuclein, a well-established component of Lewy bodies [16,17]. Indeed, LBs stained for LRRK2 and α-synuclein on adjacent sections revealed significant overlap (data not shown). In contrast, Ab2 and Ab3 recognizing the internal LRRK2_1246–1265 _and LRRK2_1838–2133_, respectively, did not recognize LBs in any of the PD cases studied (Figure 2B and 2C), as was similarly found for Ab2 in an earlier report [25]. Therefore, unlike that on Western blots, only the antibodies to the LRRK2 regions outside, but not within, the five core protein domains are able to immunostain LRRK2 localized in LBs on tissue sections. Our findings suggest that the epitopes within the folded protein domains are inaccessible under the conditions used for routine immunohistochemistry.

**Figure 3:**
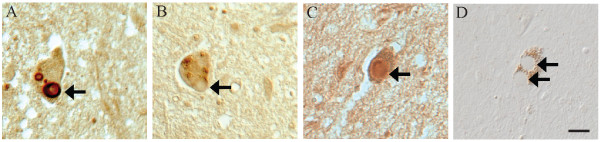
**Specificity of immunostaining for LRRK2**. Specificity of the antibodies used for immunocytochemistry was determined by performing adsorptions with the corresponding peptide sequences. Immunochemical localization of LBs (arrows) by Ab4 against LRRK2_2500–2527 _(**A**) and Ab1 against LRRK2_900–100 _(**C**) was blocked using respective peptide antigens (**B **and **D**). Adjacent sections with LB within pigmented neurons from the substantia nigra of a case of PD were used. Scale bar: 20 μm.

LRRK2 was also found to be prominent in the vasculature throughout the brainstem and cortical regions. This pattern was also found in non-diseased tissue. Interestingly, antibodies to LRRK2_1838–2133 _(Ab3, Figure 2G), as well as LRRK2_900–1000 _(Ab1, Figure 2E) and LRRK2_2500–2527 _(Ab4, Figure 2H) labelled the vasculature, whereas the antibody to LRRK2_1246–1265 _(Ab2, Figure 2F) was still negative for vessel staining. In addition, neuronal cell bodies and axons were strongly labelled by Ab4 (Figure 2H) in all cases of PD but much less intense in control cases (Figure 2L). The same pattern was also found in both PD and aged control cases with Ab1 (Figure 2E and 2I). However, no neuronal stain was seen with either Ab2 or Ab3 (Figure 2J and 2K). In contrast, small neuritic processes positive with anti-synuclein, were rarely labelled by any of the anti-LRRK2 antibodies (not shown). We confirmed that the immunostaining of LBs and other structures positive for LRRK2 was highly specific as it was mostly abolished following absorption of the primary antibodies with the corresponding peptide antigens of LRRK2 (Figure 3). Taken together, we conclude that LRRK2 within the blood vessel and neuronal structures (neuronal cell bodies and axons) shows differential regional exposure of antigenic sites as demonstrated by its distinct accessibility to different antibodies.

### Immunocytochemistry-DLB

**Figure 4:**
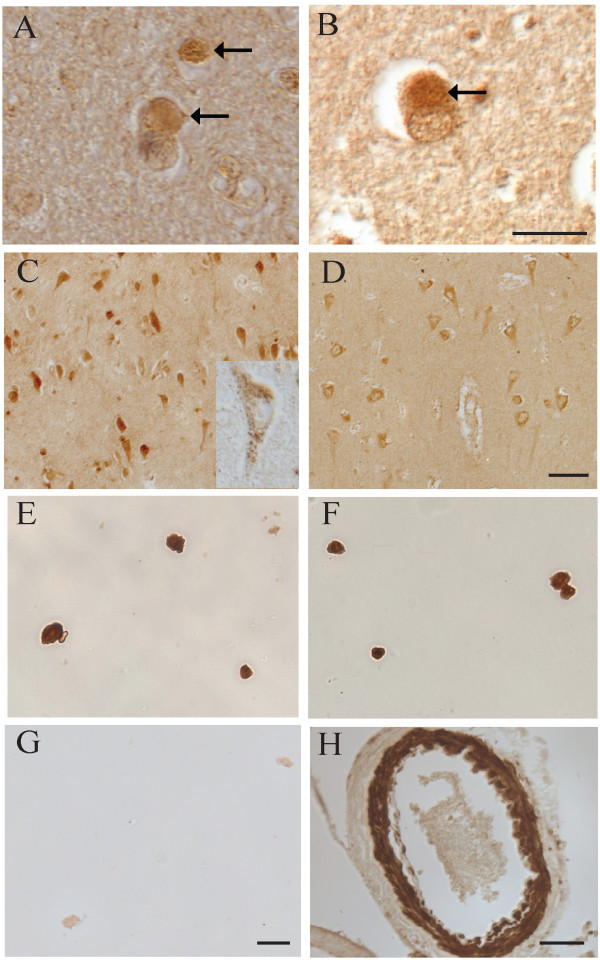
**Immunocytochemistry of LRRK2 in DLB**. Cortical LBs (arrows) in DLB were positive for LRRK2 using both Ab4 against LRRK2_2500–2527 _(**A**), and Ab1 against LRRK2_900–100 _(**B**). Neuronal cytoplasm (**C**) was also strongly labelled throughout the cortex by Ab4 and with a consistently more granular pattern by Ab1 (inset in **C**) in cases of DLB. Control cases display similar, though less intense neuronal labeling (**D**). Biochemically purified cortical LBs were strongly positive for staining by Ab4 (**E**) as well as by anti-α-synuclein (**F**), while they were unstained when omitting primary antibody (**G**). Large vessels (**H**) were also intensely labelled by Ab4. Scale bars: **A**, **B **= 25 μm; **C**, **D **= 100 μm; **E-G **= 25 μm; **H **= 50 μm.

In tissue sections from six cases of DLB, cortical LBs were also labelled by the Ab4 and Ab1 antibodies to C- and N-terminal LRRK2_2500–2527 _and LRRK2_900–1000 _regions, respectively (Figure 4A and 4B), but not by Ab2 and Ab3 to the internal regions of LRRK2_1246–1265 _and LRRK2_1838–2133_. These findings are very consistent with that for brainstem LBs of PD, confirming the notion that LBs from PD and DLB may have similar compositions despite different morphology and location. While the α-synuclein antibody stained cortical LBs exclusively, anti-LRRK2 Ab4 recognizes not only cortical LBs, but also neuronal cytoplasm (Figure 4C) more intensely than age-matched controls (Figure 4D), and vessel walls (Figure 4H). Ab1 also strongly labelled neuronal cytoplasm in these cases, but displaying a more granular pattern (inset in Figure 4C). Purified cortical LBs, biochemically isolated from DLB brains [15], were positive for staining by both anti-α-synuclein (Figure 4F) and anti-LRRK2 Ab4 (Figure 4E), whereas omitting the primary antibody revealed no labeling of the purified cortical LBs (Figure 4G). Taken together, our results strongly indicate that LRRK2 is localized to LBs both *in situ *and following isolation, and is a specific biochemical marker for PD and DLB.

## Discussion

To date, several genes with mutations linked to familial forms of PD have been identified, such as α-synuclein, parkin, PTEN-induced putative kinase 1, DJ-1, tau protein, and LRRK2. Mutations in all above except LRRK2 have been associated with early-onset or pathologically atypical forms of PD. Pathogenic mutations in LRRK2 have been recently identified in both autosomal dominant familial PD and sporadic PD [6-11,26], and are thus far the most important genetic factor for predicting late-onset familial and sporadic PD [3,27,28]. Apart from the highest prevalence among PD associated genes, LRRK2 mutations lead to a predominantly late-onset clinical phenotype that resembles idiopathic PD [12-14]. LRRK2 has thus emerged as, perhaps, the most relevant player in the pathogenesis of PD.

While there is increasing interest in the role of LRRK2 in the etiology of PD, most studies have focused on genetic predominance using DNA analysis of living donors. The paucity of reports on the normal and pathological distribution of LRRK2 protein is likely due in part to the availability of specific antibodies and the presence of exposed epitopes of LRRK2 in tissue sections. Instead, an alternative approach using in situ hybridization has been used to map mRNA expression of LRRK2 in rodent and human brains. It has been reported that mRNA of LRRK2 is expressed in dopamine-receptive (striatum) rather than dopamine-synthesizing (brainstem) regions in mice, rats and humans [29,30]. However, no difference was found between control subjects and PD cases [30]. It is likely that the mRNA expression levels may not accurately reflect the accumulation of LRRK2 protein in pathological brains.

Until now, the role of LRRK2 protein in PD pathology using immunohistochemical detection of LBs with a single antibody has thus far proved elusive. Previous studies have showed that an antiserum against the central region of LRRK2 do not recognize LBs [25] whereas antisera directed at the N-terminus [31] and C-terminus [31,32] of the protein specifically label LBs. The possible reasons for this discrepancy may be due to the difference in experimental conditions such as tissue processing and antibody specificity. Analysis of protein localization to LBs, composed of tightly packed filaments, can be inherently difficult, in particular problems with epitope masking in tissue sections. In our present study, a battery of specific antibodies directed against different regions of LRRK2 has been used for detailed analysis of serial tissue sections from PD and DLB cases. This strategy provides us an opportunity to overcome obstacles associated with differential exposure of antigenic sites of LRRK2. Of the four different antibodies tested, the two antibodies (Ab1 and Ab4) which strongly label LBs are directed against epitopes located outside the five folded domains of LRRK2. The antibody against LRRK2_1246–1265_, Ab2, with an epitope within the LRRs domain was negative for LB staining, as previously published [25], as well as negative for cell bodies and vessels. Indeed, LBs remain unstained also using an antibody against other folded domains (LRRK2_1838–2133_), Ab3, yet this antibody still recognized LRRK2 protein present in the vasculature. We postulate that LRRK2 sequences outside the folded domains (such as LRRK2_900–1000 _and LRRK2_2500–2527_) are highly exposed in LBs and most other brain structures. However, some sequences within the folded regions of LRRK2 (such as LRRK2_1838–2133_) are masked in LBs but not in brain vascular structures, whereas other sequences (such as LRRK2_1246–1265) _are masked in LBs as well as other brain components. It should be noted that all of antibodies tested have a positive immunoreactivity to LRRK2 on Western blots. However, as demonstrated in the present study, their usefulness in recognizing the respective LRRK2 epitopes in tissue sections differs widely. Our study highlights the importance of vigorous investigation in immunocytochemical analysis using multiple antibodies against different structural regions to circumvent epitope masking, especially for a large protein with a complex composition like LRRK2.

In light of the recent *in vitro *observation that LRRK2 overexpression leads to protein aggregation in cultured cells [33], it is particularly intriguing to find an apparent accumulation of LRRK2 in PD brains examined as compared to control cases. The accumulation of LRRK2 is localized to LBs, the proteinaceous inclusions, in brain regions most affected by PD (brainstem) and DLB (cortical area). Our findings are consistent with a potential gain of function for LRRK2 in disease pathogenesis, and are in line with genetic studies [3,27,28] that have found LRRK2 mutations thus far the most frequent cause of autosomal dominant PD and sporadic PD.

LRRK2 is predicted to contain several functionally important domains with multiple scaffold protein modules and signaling activities, including Ras-related GTPase and MAPKKK (mitogen-activated protein kinase kinase kinase) positioned at the top-tier of signal transduction cascades that regulate diverse arrays of cellular processes [24,34]. Hence, LRRK2 is likely to contribute to the regulatory machinery that helps maintain homeostasis of neuronal functions through its intrinsic enzymatic reactions such as GTP binding and hydrolysis, and protein phosphorylation. Recent reports have confirmed that LRRK2 indeed functions as a kinase and pathogenic mutations augment its activity in cell culture models [22,23]. Interestingly, two other proteins associated with PD, α-synuclein and parkin, are phosphoproteins localized to LBs. It is well established that α-synuclein is a major component of LBs [16-18], and selective and extensive phosphorylation of α-synuclein has been observed in both PD and DLB [19,20], and *in vitro *phosphorylation of α-synuclein has been shown to promote its aggregation [35]. Parkin is an ubiquitin ligase, also localized to LBs of PD and DLB [18]. Phosphorylation of parkin has been shown to regulate its ubiquitin ligase activity [21]. Importantly, a close interaction between LRRK2 and parkin has been recently reported [33]. Our present study has identified LRRK2 as the first signaling protein that is both genetically associated with PD and a constituent of its hallmark pathology. The co-localization of the protein kinase LRRK2 in LBs may provide a molecular mechanism underlying divergent causes associated with parkinsonism, including aberrant protein phosphorylation and subsequent protein aggregation mediated by α-synuclein and parkin. As the PD and DLB cases we investigated are sporadic and patients carrying the common LRRK2 mutations seem to share a typical late-onset disease presentation [12-14], our findings suggest a universal role of LRRK2 in etiology and pathogenesis of parkinsonism. Taken together, we conclude that LRRK2 is an essential component of LBs in PD and DLB. The expression patterns of LRRK2 in normal and pathological brains we described here will help design plausible experimental models of PD, and more importantly strategies toward productive therapeutics.

## Conclusion

We have demonstrated that LRRK2 is localized to both brainstem LBs in idiopathic PD and cortical LBs in DLB, indicating that LRRK2 is indeed an essential component of the LBs in these two neurodegenerative diseases. Our findings suggest that LRRK2 may play an important role in the Lewy body formation and disease pathogenesis of PD and DLB.

## Materials and methods

### Brain tissue

Tissue samples were obtained from the Case Medical Center and the National Institute of Child Health and Human Development (NICHD) Brain and Tissue Bank for Developmental Disorders. Brain tissue specimens were obtained postmortem from patients with histopathologically confirmed PD (n = 6; age range 53–76 years), DLB (n = 6; age range 68–85 year), and from control patients without neurological disease (n = 4; age range 42–79 years for brainstem and n = 5; age range 31–74 for neocortex samples). Sections of midbrain containing substantia nigra pars compacta were examined for PD cases, and hippocampus with adjacent cortical tissue was examined for cases of DLB. Corresponding areas from the control cases were also examined.

### LRRK2 antibodies

Affinity-purified rabbit polyclonal antibodies raised against different regions of the LRRK2 sequence were used for this study. These include Ab1 (Cat #NB300-267, Novus Biologicals, Littleton, CO) raised against LRRK2 residues between 900 and 1000 (LRRK2_900–1000_); Ab2 (Cat #AP7099b, Abgent, San Diego, CA) raised against LRRK2 residues between 1246–1265 (LRRK2_1246–1265_); Ab3 (Cat #ALX-210-928-C100, Alexis Biochemicals, San Diego, CA) raised against LRRK2 residues between 1838–2133 (LRRK2_1838–2133_); and Ab4 (Cat #NB300-268, Novus Biologicals, Littleton, CO) raised against LRRK2 residues 2500–2527 (LRRK2_2500–2527_).

### Western blotting

Recombinant LRRK2 was overexpressed in human embryonic kidney 293T cell line or human neuroblastoma M17 cell line, following transient transfection with a mammalian expression vector containing human LRRK2 cDNA (pCMV6-XL4/LRRK2; Origene Techonologies, Rockville, MD). Transfection was performed using the Lipofectamine 2000 reagent (Invitrogen, Carlsbad, CA), and cell lysates were collected in lysis buffer (1% Triton X-100 in phosphate-buffered saline with the Complete protease inhibitors (Roche Applied Science, Indianapolis, IN)) after 48 h. Brain homogenates prepared from frozen brainstem samples from cases of Parkinson disease and age matched controls (10% w/v) were made in a buffer containing 20 mM Tris, pH 7.5, 150 mM NaCl, 0.5% NP 40, 0.5% Na deoxycolate, and the Complete protease inhibitors (Roche). The protein concentration was determined with a DC protein assay kit (Bio-Rad, Hercules, CA). An equivalent of 100 μg (brain homogenates) or 10 μg (cell lysates) of proteins was used for Western blot analysis. Cell lysates or brain homogenates were boiled for 10 minutes in sodium dodecyl sulfate (SDS) sample buffer (3% SDS; 2 mM EDTA; 10% glycerol; 50 mM Tris-HCl, pH 6.8). Proteins were separated by SDS-polyacrylamide gel electrophoresis (SDS-PAGE, 6% gels), and transferred to polyvinylidene difluoride membranes. The membranes were blocked with 10% nonfat milk prepared in Tris-buffered saline containing 0.1% Tween 20 (TBST) and then incubated for 16 hours at 4°C with anti-LRRK2 antibodies at appropriate dilutions (Ab1, 1:3,000; Ab2, 1:500; Ab3, 1:5,000; and Ab4, 1:6,000). As an absorption control, an antibody was incubated with its peptide antigen at the ratio of 1:10 (w/w) for 1 h at room temperature prior to final dilution. Following incubation with a horseradish peroxidase conjugated anti-rabbit secondary antibody (Amersham Biosciences, Piscataway, NJ), LRRK2 was visualized by enhanced chemiluminescence (ECL plus kit; Amersham Biosciences) according to the manufacturer's instructions.

### Immunocytochemistry

Brain tissue was fixed in formalin and was subsequently dehydrated through graded ethanol and xylene solutions and embedded in paraffin. Six-micron thick microtome sections were prepared and placed on silane-coated slides. Following hydration, sections were immunostained by the peroxidase-antiperoxidase procedure using anti-LRRK2 antibodies at a final dilution of 1:100 to 1:200. Adjacent sections were also immunostained with an antibody to α-synuclein (Chemicon, Temecula, CA) to locate the Lewy bodies. Purified cortical Lewy bodies were prepared from brain tissue affected by DLB as described [15], and were subsequently dried onto silane-coated glass slides for immunostaining of α-synuclein and LRRK2. To verify the specificity of immunolabeling of LRRK2, primary antibody was omitted as a negative control. In addition, adsorption control experiments were performed by incubating peptides (Novus Biologicals) corresponding to LRRK2_900–1000 _and LRRK2_2500–2527 _with corresponding antibodies on some cases. The diluted primary antibodies were incubated for 16 hours at 4°C with the peptides (0.2 mg/ml) and used for immunocytochemistry.

## Competing interests

The author(s) declare that they have no competing interests.

## Authors' contributions

SGC and XZ designed overall study, and contributed to the data interpretation and preparation of the manuscript. AB and SLS undertook immunostaining of tissue sections and contributed to manuscript preparation. QY provided technical assistance for Western blot assays. GI and TI provided purified cortical Lewy bodies and contributed to manuscript preparation. MAS and GP contributed to the data interpretation and preparation of the manuscript.
